# Effect of altitudes on serum parameters, metabolome, and gut microbiota in yaks on the Qinghai-Tibet Plateau

**DOI:** 10.1128/spectrum.02549-25

**Published:** 2025-12-18

**Authors:** Yining Xie, Yangji Cidan, Zhuoma Cisang, Renzeng Ciwang, Guifang Liu, Dan Wu, Duoji Cideng, Jiacuo Chilie, Jilam Kang, Yanbin Zhu, Wangdui Basang

**Affiliations:** 1 Institute of Animal Husbandry and Veterinary Medicine, Xizang Academy of Agricultural and Animal Husbandry Sciences 232815, Lhasa, China; 2 State Key Laboratory of Animal Nutrition and Feeding, Institute of Animal Science, Chinese Academy of Agricultural Sciences 12661, Beijing, China; 3 Precision Livestock and Nutrition Unit, TERRA Teaching and Research Centre, Gembloux Agro-Bio Tech, University of Liège 82209, Gembloux, Belgium; 4 Nie Rong County Agriculture and Animal Husbandry Comprehensive Service Center, Nagqu City, China; 5 Institute of Scientific and Technical Information of Xizang Autonomous Region, Lhasa, China; Ocean University of China, Qingdao, Shandong, China

**Keywords:** altitude, serum parameters, plasma metabolome, fecal microbiota

## Abstract

Yaks (*Bos grunniens*), native to the Qinghai-Tibet Plateau, have evolved extraordinary physiological resilience to chronic hypoxia, cold, and nutritional scarcity. However, the integrated metabolic and microbial mechanisms underlying these adaptations remain poorly defined. Here, a comprehensive multi-omics analysis was performed on thirty grazing heifer yaks (2.5 years old) from three altitudes—3,600 m (low altitude [LA]), 4,000 m (middle altitude [MA]), and 4,500 m (high altitude [HA])—to investigate how altitude affects host physiology, metabolism, and gut microbial ecology. Increasing altitude significantly reduced serum total protein, globulin, blood urea nitrogen, and alkaline phosphatase, indicating suppressed anabolic metabolism and nitrogen-sparing strategies. Antioxidant capacity (total superoxide dismutase, total antioxidant capacity) and pro-inflammatory cytokines (interleukin-2 [IL-2], IL-6, tumor necrosis factor-α, interferon-γ) increased (*P* < 0.05), while glutathione peroxidase, IL-4, IL-10, growth hormone, insulin-like growth factor-1, and growth hormone-releasing hormone declined (*P* < 0.05), reflecting energy reallocation from growth toward antioxidation and immune maintenance under hypoxia. Plasma metabolomics revealed distinct altitude-dependent reprogramming, with enrichment of retinol metabolism at 4,000 m and α-linolenic acid metabolism, tricarboxylic acid (TCA) cycle, and branched-chain amino acid biosynthesis at 4,500 m. These pathways link lipid remodeling, oxidative balance, and oxygen utilization. The gut microbiota displayed altitude-specific shifts, characterized by enrichment of *Christensenellaceae_R-7_group* and *Monoglobus* and reduced *UCG-005* and *Rikenellaceae_RC9_gut_group*, accompanied by lower fecal volatile fatty acids (*P* < 0.05). Correlation analyses confirmed tight associations between fermentative taxa and volatile fatty acids production. Collectively, our results establish a serum–metabolome–microbiota axis as a central mechanism supporting yak adaptation to high altitude.

**IMPORTANCE:**

This study demonstrates that the gut microbiota plays a crucial role in how yaks adapt to high-altitude hypoxia. Rising altitude not only alters the composition of gut microbes but also shifts their metabolic activity toward improving fermentation efficiency and antioxidant capacity. These microbial changes are closely linked with host metabolism, forming a coordinated serum–metabolome–microbiota network that helps maintain energy balance and immune stability when oxygen is limited. The enrichment of retinol and α-linolenic acid metabolism as altitude-responsive pathways further highlights the metabolic interplay between host and microbes in supporting physiological resilience. Overall, our findings show that microbial flexibility and metabolic cooperation are key factors enabling ruminants to survive in extreme environments, providing a scientific basis for microbiome-informed strategies to enhance yak health and productivity on the Qinghai-Tibet Plateau.

## INTRODUCTION

The yak (*Bos grunniens*) is a representative domestic animal inhabiting the Qinghai-Tibet Plateau and the Himalayan region, capable of surviving in extreme environments characterized by hypoxia, severe cold, and intense ultraviolet radiation (1, 2). Its milk, meat, and fiber products are vital resources for local herders, supporting both subsistence and livelihoods (3). With increasing altitude, oxygen partial pressure decreases, temperature fluctuations intensify, and ultraviolet radiation strengthens—all of which can markedly affect physiological functions, including energy metabolism, immune responses, hormonal regulation, and overall health.

Serum biochemical parameters comprehensively reflect the metabolic and physiological status of animals, including energy and protein metabolism, liver and kidney function, oxidative stress, and immune balance. These indices are valuable indicators for assessing the physiological adaptability of animals to different environmental conditions. High-altitude hypoxia not only challenges energy metabolism but also imposes persistent stress on the immune system, triggering complex immune regulatory responses (4, 5). Populations and animals living long-term in high-altitude environments often exhibit stronger immune adaptability, which is considered an evolutionary strategy to cope with environmental stress and potential pathogenic threats (6, 7). Moreover, hormones related to growth and metabolism play essential roles in regulating energy allocation, metabolic balance, and tissue development, and their variations reflect the organism’s adaptive adjustments to high-altitude environments (8). In addition, rising altitude profoundly influences the composition and function of the host gut microbiota. Gut microorganisms contribute to host adaptation by modulating the production of short-chain fatty acids (VFAs) and other metabolites, thereby participating in energy metabolism and immune regulation that are crucial for maintaining host health (9, 10).

Based on these considerations, we hypothesize that with increasing altitude, yaks achieve high-altitude adaptation through the coordinated regulation of physiological metabolism, immune response, hormonal balance, and gut microbial community structure. To verify this hypothesis, this study systematically investigated serum biochemical characteristics, metabolic profiles, and gut microbial composition of yaks from different altitudes on the Qinghai-Tibet Plateau, aiming to elucidate the integrated mechanisms underlying yak adaptation to high-altitude environments. The findings will provide scientific insights into the physiological mechanisms of high-altitude adaptation in yaks and offer a theoretical basis for sustainable breeding and health management of yak populations in plateau regions.

## MATERIALS AND METHODS

### Experimental design and sample collection

The experiment was carried out in September 2024. Thirty grazing heifer yaks around 2.5 years old were randomly selected from natural pastures at an altitude of 3,600 m in Shannan City, Nedong District, Suozhu Township (N28°12′, E92°21′, low altitude group, LA), 4,000 m Linzhou County, Songpan Township (N29°45′, E90°51′, middle altitude group, MA), and 4,500 m in Nyerong County, Seqing Township (32.06°N, 92.18°E, high altitude group, HA). After all the yaks had been fasted for 24 h, tail root blood sampling was performed on them using vacuum blood collection tubes with or without heparin sodium. The samples were stored away from light for half an hour and then centrifuged at 3,000 revolutions per minute for 15 min to collect plasma and serum. Fresh feces were obtained through the rectal massage method. All the samples were temporarily stored with dry ice and then transferred to a −80°C freezer for subsequent analysis.

### Determination of serum indicators

In this study, a comprehensive analysis of biochemical indicators in serum was conducted using various diagnostic kits. The specific methods employed include the Dumas method diagnostic kit for measuring serum total protein (TP); the BCG method diagnostic kit for measuring serum albumin; the blood urea nitrogen (BUN) method diagnostic kit for measuring BUN; the CHOD-POD method diagnostic kit for measuring serum total cholesterol (TC); the GPO-PAP method diagnostic kit for measuring triglycerides (TG); the glucose oxidase method diagnostic kit for measuring glucose; the CC kinetic method diagnostic kit for measuring serum alanine aminotransferase (ALT); the IFCC kinetic method diagnostic kit for measuring aspartate aminotransferase (AST); and the dedicated ALP detection method diagnostic kit for measuring alkaline phosphatase (ALP). Additionally, specific diagnostic kits were used to measure the levels of total superoxide dismutase (T-SOD), total antioxidant capacity (T-AOC), malondialdehyde, and glutathione peroxidase (GSH-Px) in serum. In addition, to accurately determine the levels of immune and growth-related indicators in yak serum, this study utilized bovine-specific enzyme-linked immunosorbent assay kits to quantify immunoglobulins IgA, IgM, and IgG, as well as various cytokines. These cytokines include interleukin-2 (IL-2), IL-4, IL-6, IL-10, tumor necrosis factor-α (TNF-α), interferon-γ (IFN-γ), growth hormone (GH), insulin-like growth factor-1 (IGF-1), growth hormone-releasing hormone (GHRH), and growth hormone-inhibiting hormone (GHIH).

### Plasma metabolome

Extraction of metabolites involved the addition of 50 mg of a solid sample into a 2 mL centrifuge tube that contained a 6 mm grinding bead. This was followed by the introduction of 400 µL of extraction solution (composed of methanol and water in a 4:1 ratio, with a concentration of 0.02 mg/mL). The samples were ground for 6 min at −10°C and then subjected to ultrasonication at 5°C for 30 min. Afterward, the samples were maintained at −20°C for an additional 30 min before being centrifuged at 4°C and 13,000 × *g* for 15 min. The resulting supernatant was subsequently placed into an injection vial for analysis via LC-MS/MS. A pooled quality control (QC) sample was generated by combining equal volumes from all individual samples, which was injected regularly to ensure the stability of the analytical results. The LC-MS/MS analysis utilized a Thermo UHPLC-Exploris 240 system equipped with an ACQUITY HSS T3 column. The mobile phases included 0.1% formic acid in water and acetonitrile (95:5), as well as 0.1% formic acid in a mixture of acetonitrile, isopropanol, and water (47.5:47.5:5). Gradient elution was carried out with varying profiles for both positive and negative ion modes, while the mass spectrometric conditions were fine-tuned using an ESI source, with adjustments made to temperature, gas flow rates, and voltages. Data acquisition was executed in Data Dependent Acquisition mode within a mass range of 70–1,050 m/z.

### Fecal volatile fatty acids

A stock solution A was prepared by mixing six VFAs—acetic, propionic, butyric, isobutyric, isovaleric, and valeric—in 9,840 µL of HPLC-grade butanol. Stock solution B was created by adding 10 µL of 2-ethylbutyric acid to 9,990 µL of butanol as an internal standard. These stock solutions were further diluted into seven working solutions for GC-MS analysis. For sample preparation, 25 mg of fecal samples were processed by adding 500 µL of water containing 0.5% phosphoric acid. The samples were then subjected to freeze-grinding (50 Hz, 3 min × 2), sonication (10 min), and centrifugation (4°C, 13,000 × *g*, 15 min). The supernatant (200 µL) was extracted with 200 µL of butanol containing 10 µg/mL 2-ethylbutyric acid, followed by vortexing (10 s), sonication (10 min), and another centrifugation (4°C, 13,000 × *g*, 5 min). The final supernatant was transferred to vials for GC-MS analysis. The GC-MS analysis was performed using an Agilent 8890B-5977B/7000D instrument equipped with an HP FFAP column and helium as the carrier gas. The temperature program was set to start at 80°C, increase to 120°C at a rate of 20°C/min, then to 160°C at 5°C/min, and finally hold at 220°C for 3 min. The mass spectrometry conditions included an EI ion source with temperatures set at 230°C for the ion source and 150°C for the quadrupole, and data acquisition was performed in SIM mode. To ensure system stability and repeatability, QC samples were inserted every 10 samples, with relative standard deviation (RSD) values for target compounds required to be below 15%.

### Fecal microbiota

Genomic DNA in 30 fecal samples was isolated from yak fecal using the E.Z.N.A. Soil DNA Kit (Omega Bio-tek, Norcross, GA, USA) and checked via 1% agarose gel electrophoresis (8). The V3–V4 region of the bacterial 16S rRNA gene was then amplified with primers 338F (5′-ACTCCTACGGGAGGCAGCAG-3′) and 806R (5′-GGACTACHVGGGTWTCTAAT-3′). The amplicons were sequenced on the Illumina MiSeq PE300 platform. Data analysis was performed using Uparse (v7.0) to cluster sequences into operational taxonomic units (OTUs) at a 97% similarity threshold and remove chimeric sequences. Taxonomic classification was conducted using the Ribosomal Database Project Classifier (v2.11) with the 16S rRNA database, applying a confidence threshold of 0.7.

### Data analysis

The data matrix was uploaded to the Majorbio Cloud Platform for analysis. It was preprocessed by removing variables with missing values using the 80% rule; imputing missing values with the minimum value; normalizing with total sum normalization; removing variables with RSD >30% in QC samples; and log10-transforming the data to obtain the final matrix for analysis. Principal component analysis (PCA) and orthogonal partial least squares discriminant analysis (OPLS-DA) were performed using the ropls package in R, with sevenfold cross-validation to assess model stability. To elucidate the metabolic pathways in our study, we utilized the Kyoto Encyclopedia of Genes and Genomes (KEGG) Pathway Database (11). This database offers detailed and comprehensive information on metabolic pathways and associated biological processes. The relevant data were obtained from the KEGG database (https://www.kegg.jp/) and subsequently processed as detailed in the subsequent sections. Differential metabolites were identified based on VIP >1 from the OPLS-DA model and *P* <0.05 from Student’s *t*-test. Pathway analysis was conducted using the KEGG database, and pathway enrichment was performed with scipy stats in Python, identifying relevant biological pathways through Fisher’s exact test. The bioinformatics analysis was conducted using Usearch software through the Majorbio Cloud Platform, clustering OTUs at a 97% similarity level for statistical analysis. Alpha diversity (Ace, Chao1, Shannon) was calculated using Mothur, and beta diversity (PCoA) was analyzed with Qiime 2. Microbial abundances at phylum and genus levels were compared using R and Python packages (Wilcoxon rank sum test). Differential taxa were identified using linear discriminant analysis effect size (LEfSe) with a Kruskal-Wallis alpha value of 0.05 and an LDA score threshold of 3.5. A Pearson correlation analysis was performed using the Pheatmap package in R, with significance set at *P* ≤ 0.05.

Before conducting statistical analysis, the normality and homogeneity of variance of the data were checked using the test procedures of JMP software (JMP, version 10; SAS Institute Inc., Cary, NC). If *P* < 0.05, it is considered significant, and if 0.05 ≤ *P* ≤ 0.10, it may indicate a trend.

## RESULTS

### Serum biochemical indicators

Compared with the low altitude group, as the altitude increased, the levels of total TP, GLB, BUN, ALT, and ALP in the serum of yaks in the MA group and the HA group decreased significantly (*P* < 0.05, Table 1). Meanwhile, the levels of TG in the serum of yaks in the LA group and the MA group were significantly lower than those in the HA group (*P* < 0.05). Interestingly, the MA group had significantly higher TC but lower AST levels compared to the other two groups (*P* < 0.05).

**TABLE 1:** Effect of altitude on biochemical indicators of yak serum^*b*^

Items	Treatment^*a*^				
	LA	MA	HA	SEM	*P* value
Total protein, g/L	74.66^a^	70.96^a^	63.39^b^	2.42	0.011
Albumin, g/L	43.35	44.65	40.42	1.41	0.1171
Globulin, g/L	30.80^a^	26.68^b^	24.15^b^	1.27	0.0047
Urea nitrogen, mmol/L	6.64^a^	4.76^b^	4.15^b^	0.29	<0.0001
Total cholesterol, mmol/L	2.86^b^	4.66^a^	2.58^b^	0.33	0.0004
Triglycerides (TG), mmol/L	1.36^b^	1.30^b^	1.49^a^	0.03	0.0004
Glucose, mmol/L	8.13	4.79	4.14	0.32	0.1065
Alanine aminotransferase, U/L	108.33^a^	93.38^b^	79.88^c^	2.84	<0.0001
Aspartate aminotransferase, U/L	41.43^a^	26.68^b^	39.63^a^	3.01	0.0042
Alkaline phosphatase, U/L	166.88^a^	155.25^a^	117.88^b^	11.72	0.0196

^*a*^ LA, low altitude yak group; MA, middle altitude yak group; HA, high altitude yak group, *n *= 10.

^*b*^ a–c: values within a row that do not share a common superscript differ significantly (*P* < 0.05).

### Serum antioxidant indicators

When compared with the low-altitude group, as the altitude increased, the levels of T-SOD and T-AOC in the serum of yaks in both the MA group and the HA group increased significantly (*P* < 0.05, Table 2). Conversely, the enzymatic activity of GSH-Px decreased significantly (*P* < 0.05).

**TABLE 2:** Effect of altitude on antioxidant indicators of yak serum^*b*^

Items	Treatment^*a*^				
	LA	MA	HA	SEM	*P* value
Total superoxide dismutase, U/mL	74.66^a^	70.96^a^	63.39^b^	2.42	0.011
Total antioxidant capacity, U/mL	108.33^a^	93.38^b^	79.88^c^	2.84	<0.0001
Malondialdehyde, nmol/mL	41.43^a^	26.68^b^	39.63^a^	3.01	0.0042
Glutathione peroxidase, U/mL	166.88^a^	155.25^a^	117.88^b^	11.72	0.0196

^*a*^ LA, low altitude yak group; MA, middle altitude yak group; HA, high altitude yak group, *n* = 10.

^*b*^ a–c: values within a row that do not share a common superscript differ significantly (*P* < 0.05).

### Serum immune indicators

With increasing altitude, the levels of immunoglobulins (IgA, IgM, and IgG), interleukins (IL-2, IL-6, and TNF-α), and IFN-γ in the serum of yaks in the MA and HA groups increased significantly (*P* < 0.05; Table 3). In contrast, the levels of IL-4 and IL-10 decreased significantly in the MA and HA groups (*P* < 0.05).

**TABLE 3:** Effect of altitude on immune indicators of yak serum^*b*^

Items	Treatment^*a*^				
	LA	MA	HA	SEM	*P* value
Immunoglobulin A, g/L	1.07^c^	1.19^b^	1.28^a^	0.01	<0.0001
Immunoglobulin M, g/L	0.90^c^	1.00^b^	1.08^a^	0.01	<0.0001
Immunoglobulin G, g/L	4.74^c^	5.41^b^	5.82^a^	0.05	<0.0001
Interleukin-2, pg/mL	183.70^c^	233.96^b^	286.78^a^	3.60	<0.0001
Interleukin-4, pg/mL	74.59^a^	65.23^b^	55.24^c^	0.67	<0.0001
Interleukin-6, pg/mL	99.11^c^	138.20^b^	173.65^a^	3.38	<0.0001
Interleukin-10, pg/mL	257.27^a^	214.14^b^	177.75^c^	2.59	<0.0001
Tumor necrosis factor α, pg/mL	109.50^c^	139.45^b^	171.39^a^	2.65	<0.0001
Interferon-γ, pg/mL	870.38^c^	1,134.49^b^	1,437.88^a^	22.18	<0.0001

^*a*^ LA, low altitude yak group; MA, middle altitude yak group; HA, high altitude yak group, *n *= 10.

^*b*^ a–c: values within a row that do not share a common superscript differ significantly (*P* < 0.05).

### Serum hormonal indicators

Compared with the LA group, the levels of GH, IGF-1, and GHRH in the serum of yaks in the MA and HA groups decreased significantly (*P* < 0.05; Table 4). Meanwhile, the concentration of GHIH increased significantly with increasing altitude (*P* < 0.05).

**TABLE 4:** Effect of altitude on growth hormone axis indicators of yak serum^*b*^

Items	Treatment^*a*^				
	LA	MA	HA	SEM	*P* value
Growth hormone, ng/mL	9.77^a^	7.68^b^	6.10^c^	0.23	<0.0001
Insulin-like growth factor-1, ng/mL	231.55^a^	185.94^b^	147.12^c^	5.60	<0.0001
Growth hormone-releasing hormone, pg/mL	24.51^a^	20.88^b^	16.86^c^	0.42	<0.0001
Growth hormone-inhibiting hormone, pg/mL	122.38^c^	153.63^b^	193.68^a^	4.30	<0.0001

^*a*^ LA, low altitude yak group; MA, middle altitude yak group; HA, high altitude yak group, *n *= 10.

^*b*^ a–c: values within a row that do not share a common superscript differ significantly (*P* < 0.05).

### Fecal volatile fatty acids

As shown in Table 5, the concentrations of acetic acid, propionic acid, isobutyric acid, butyric acid, isovaleric acid, and valeric acid in the feces of yaks in the MA and HA groups were significantly lower than those in the LA group (*P* < 0.05).

**TABLE 5:** Effect of altitude on fecal volatile fatty acids of yak^*b*^

Items	Treatment^*a*^				
	LA	MA	HA	SEM	*P* value
Acetic acid, ug/mg	2,983.43^a^	1,241.95^b^	1,380.66^b^	198.21	<0.0001
Propanoic acid, ug/mg	687.00^a^	242.34^b^	268.23^b^	47.86	<0.0001
Isobutyric acid, ug/mg	103.27^a^	66.84^b^	51.66^b^	8.99	0.0012
Butanoic acid, ug/mg	472.32^a^	179.15^b^	171.38^b^	39.83	<0.0001
Isovaleric acid, ug/mg	43.65^a^	25.36^b^	20.21^b^	4.72	0.0041
Valeric acid, ug/mg	79.35^a^	30.61^b^	29.60^b^	6.60	<0.0001

^*a*^ LA, low altitude yak group; MA, middle altitude yak group; HA, high altitude yak group, *n *= 10.

^*b*^ a–c: values within a row that do not share a common superscript differ significantly (*P* < 0.05).

### Plasma metabolome

**Fig 1:**
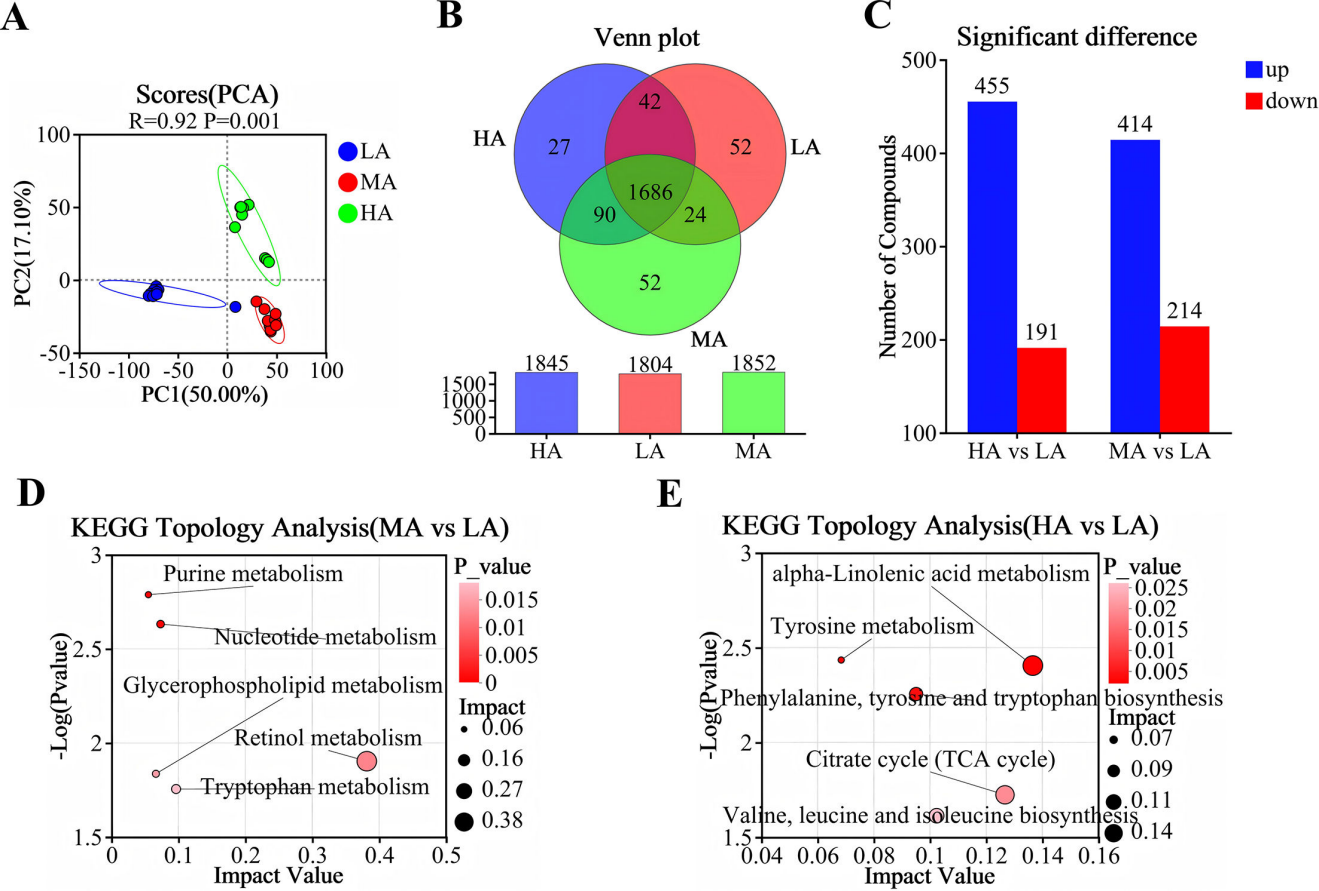
Plasma metabolome analysis diagrams. (**A**) PCA score plot; (**B**) Venn plot; (**C**) volcano plot of differential metabolites (MA vs LA); (**D**) KEGG topological analysis diagram (MA vs LA); (**E**) volcano plot of differential metabolites (HA vs LA); KEGG topological analysis diagram (HA vs LA). LA, low altitude yak group; MA, middle altitude yak group; HA, high altitude yak group, *n = 10*.

PCA revealed clear separations in the plasma metabolic profiles of yaks from different altitudes (R = 0.92, *P* < 0.01), demonstrating the scientific validity and reliability of the subsequent analyses (Fig. 1A). Venn diagram analysis showed that 1,804, 1,852, and 1,845 metabolites were detected in the plasma of yaks from the LA group, MA group, and HA group, respectively, with 1,686 metabolites shared across all three groups (Fig. 1B). Compared with the LA group, 414 metabolites were upregulated and 214 were downregulated in the MA group, whereas 455 metabolites were upregulated and 191 were downregulated in the HA group (Fig. 1C). Pathway enrichment analysis further identified the top five significantly enriched pathways in the MA group, including *retinol metabolism*, *tryptophan metabolism*, *nucleotide metabolism*, *glycerophospholipid metabolism*, and *purine metabolism* (Fig. 1D). In contrast, the top five enriched pathways in the HA group were *α-linolenic acid metabolism*, *citrate cycle (TCA cycle*), *valine, leucine, and isoleucine biosynthesis*, *phenylalanine, tyrosine, and tryptophan biosynthesis*, and *tyrosine metabolism* (Fig. 1E).

### Fecal microbiota

**Fig 2:**
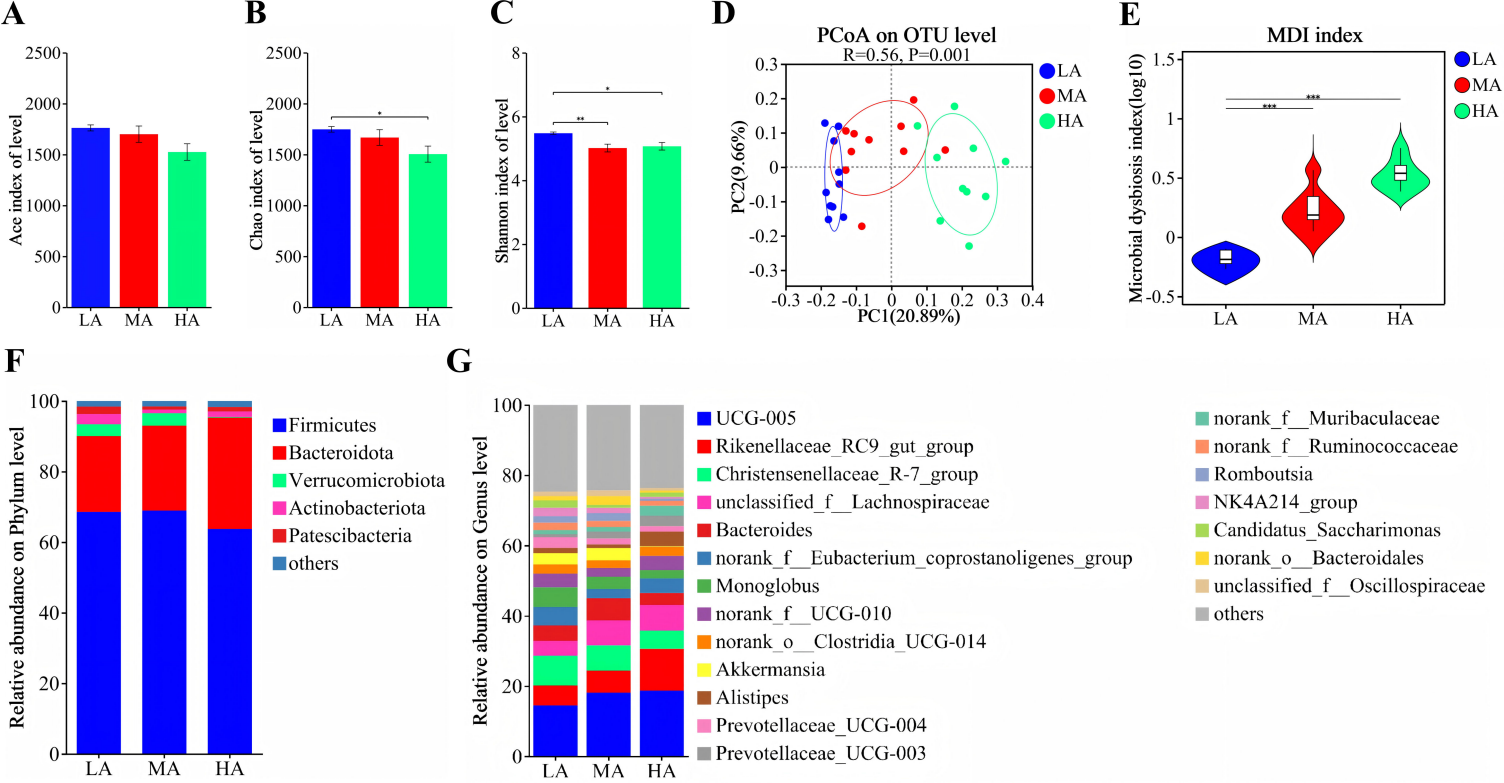
The diversity of fecal microbiota. (**A**) Ace index; (**B**) *Chao1* index; (**C**) Shannon index; (**D**) PCoA score plot; (**E**) MDI; (**F**) microbial compositions at the phylum level; (**G**) microbial compositions at the genus level. *, ** and *** indicate *P* < 0.05, *P* < 0.01, and *P* < 0.001, respectively.

**Fig 3:**
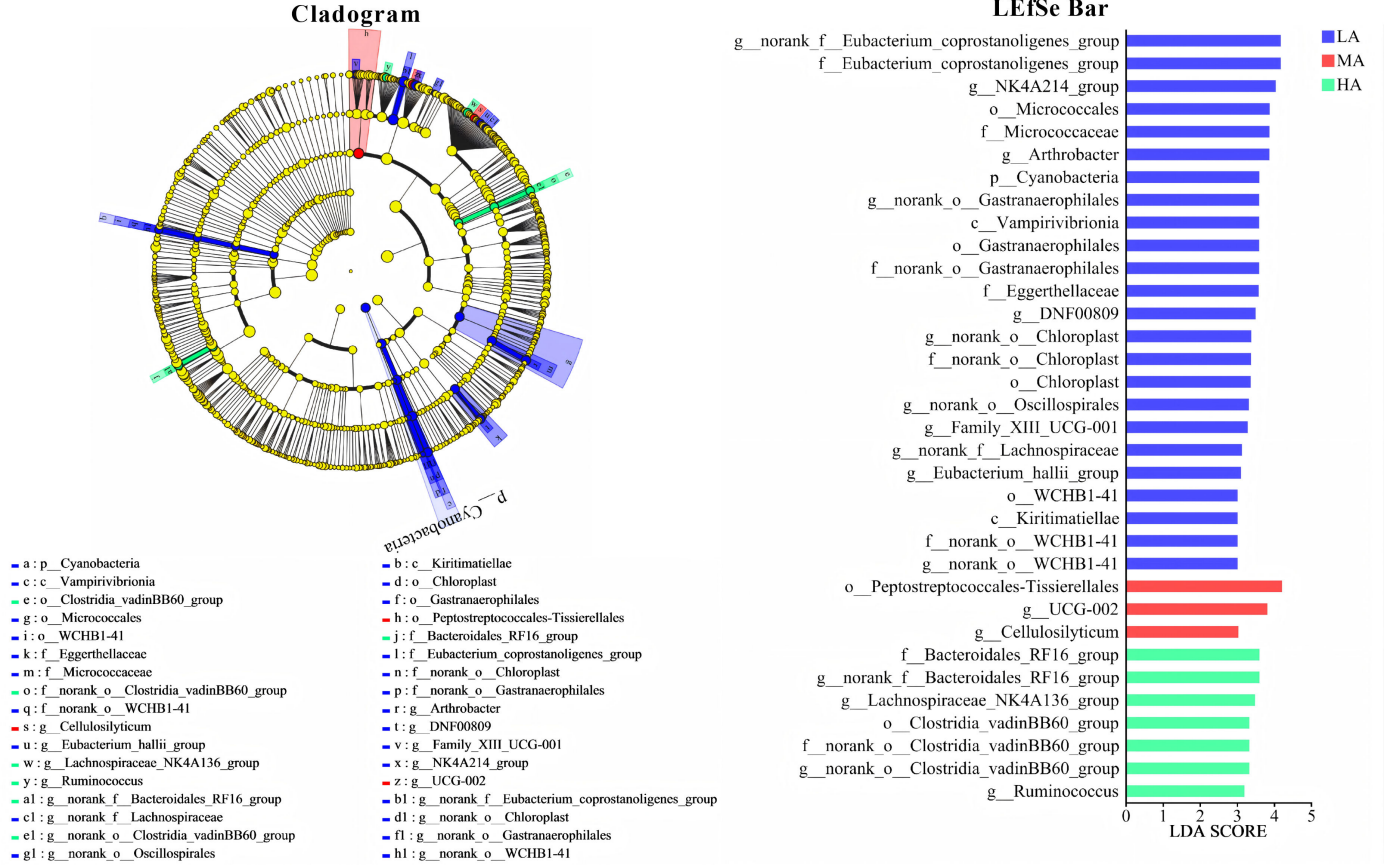
LEfSe hierarchical taxonomic tree at multiple levels.

**Fig 4:**
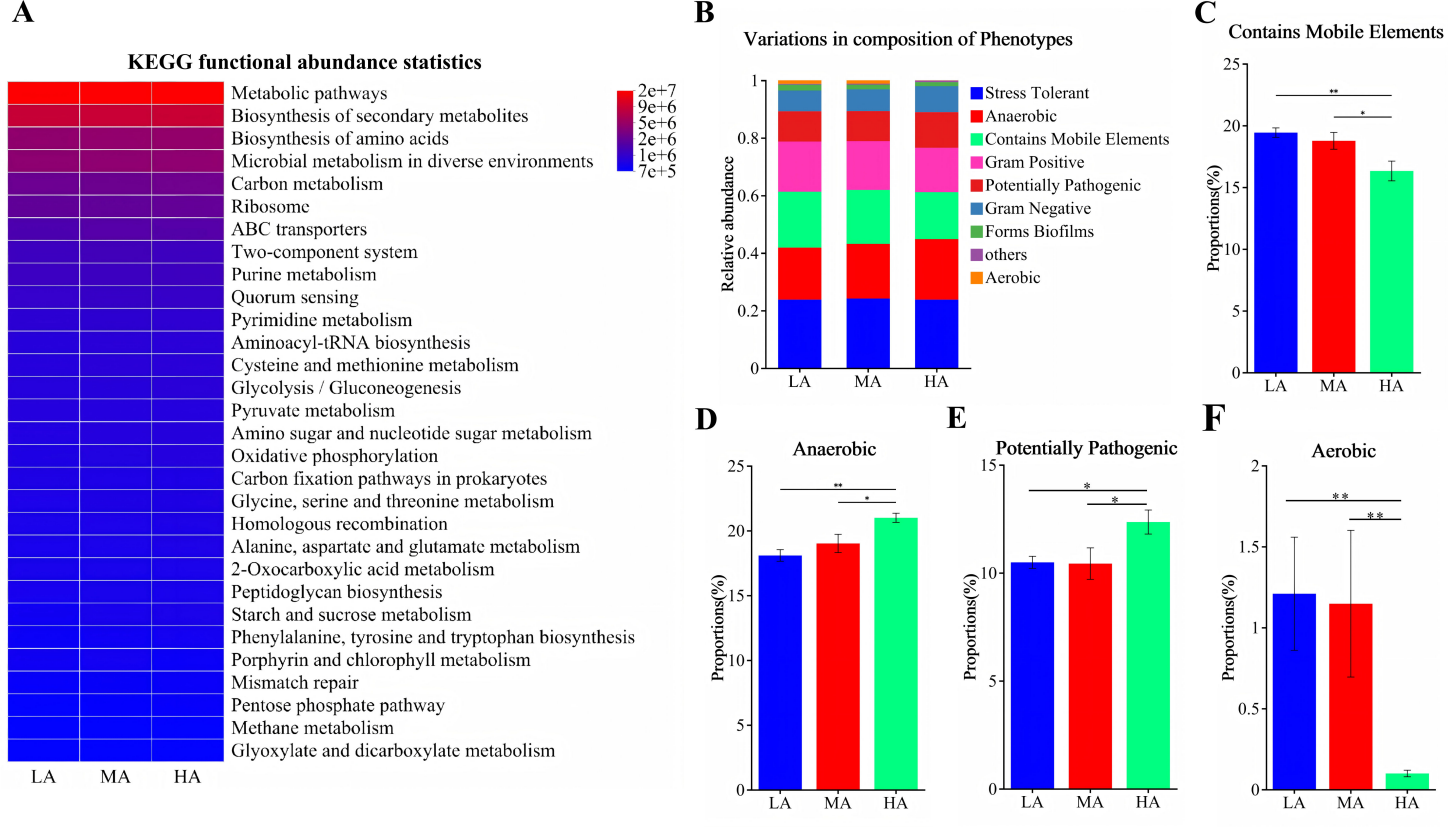
Functional prediction of microbial communities. (**A**) Functional prediction based on PICRUSt2; (**B**) phenotypic prediction using BugBase; (**C**) relative abundance of *Contains Mobile Elements* phenotype; (**D**) relative abundance of *Anaerobic* phenotype; (**E**) relative abundance of *Potentially Pathogenic* phenotype; and (**F**) relative abundance of *Aerobic* phenotype. * and ** indicate *P* < 0.05 and *P* < 0.01, respectively.

A total of 30 fecal samples from yaks across three altitude groups were analyzed to assess microbial diversity. Altitude significantly affected the α-diversity of the fecal microbiota (Fig. 2A through C), as evidenced by a reduced Shannon index in the MA group and lower Chao1 and Shannon indices in the HA group. In addition, altitude markedly influenced the β-diversity of the fecal microbiota, as indicated by distinct PCoA clustering among groups (R = 0.56, *P* = 0.001; Fig. 2D). The microbial dysbiosis index (MDI) also showed a significant elevation in the MA and HA groups compared with the LA group (Fig. 2E), suggesting increased microbial imbalance with rising altitude. At the phylum level, altitude significantly decreased the relative abundance of Verrucomicrobiota (Fig. 2F), as well as Actinobacteriota in the HA group and Actinobacteriota in both the MA and HA groups (Fig. S1A and B). At the genus level, among the top 20 taxa (Fig. 2G), 13 genera exhibited significant differences across altitudes (Fig. S1C through O). Compared with the LA group, the MA group showed a higher relative abundance of *UCG-005*, while *norank_f__Eubacterium_coprostanoligenes_group, Christensenellaceae_R-7_group*, *Monoglobus*, *norank_f__UCG-010*, *Prevotellaceae_UCG-004*, and *NK4A214_group* were significantly reduced. In the HA group, the relative abundances of *UCG-005*, *Rikenellaceae_RC9_gut_group*, *Alistipes*, and *norank_f__Muribaculaceae* were significantly increased, whereas *norank_f__Eubacterium_coprostanoligenes_group, Christensenellaceae_R-7_group*, *Monoglobus*, *Akkermansia*, *Prevotellaceae_UCG-004*, *norank_f__Ruminococcaceae*, *Romboutsia*, and *NK4A214_group* were significantly decreased. LEfSe analysis (LDA score > 3) identified 34 differential taxa ranging from phylum to genus level among the three altitude groups (Fig. 3). Functional prediction based on PICRUSt2 indicated that the altered fecal microbiota were mainly enriched in metabolic pathways, biosynthesis of secondary metabolites, biosynthesis of amino acids, microbial metabolism in diverse environments, and carbon metabolism (Fig. 4A). Moreover, BugBase phenotypic prediction revealed that, relative to the LA group, the HA group showed significantly lower proportions of microorganisms classified as Contains Mobile Elements and Aerobic, but significantly higher proportions of Anaerobic and Potentially Pathogenic phenotypes (Fig. 4B through F).

### Correlation analysis

**Fig 5:**
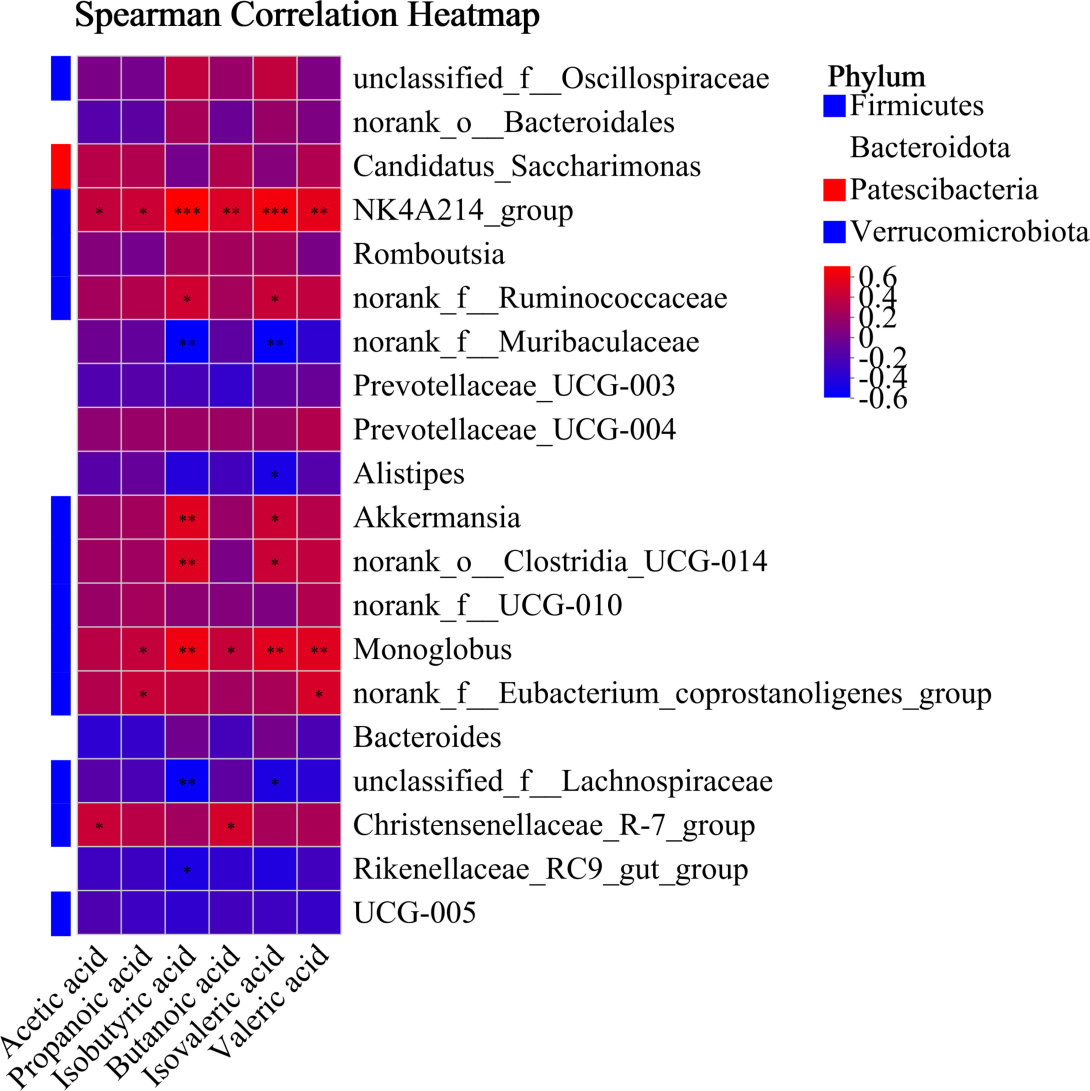
Spearman’s correlation analysis between VAFs and the top 20 microbial genera. *, **, and *** indicate *P* < 0.05, *P* < 0.01, and *P* < 0.001, respectively.

Spearman’s correlation analysis was conducted between six VFAs and the top 20 microbial genera(Fig. 5). The results showed that acetic acid was positively correlated with *NK4A214_group* (R = 0.37, *P* = 0.05) and *Christensenellaceae_R-7_group* (R = 0.39, *P* = 0.03). Propanoic acid exhibited positive correlations with *NK4A214_group* (R = 0.40, *P* = 0.03), *Monoglobus* (R = 0.37, *P* = 0.04), and *norank_f__Eubacterium_coprostanoligenes_group* (R = 0.38, *P* = 0.04). Isobutyric acid showed significant positive correlations with *NK4A214_group* (R = 0.63, *P* < 0.01), *norank_f__Ruminococcaceae* (R = 0.42, *P* = 0.02), *Akkermansia* (R = 0.49, *P* = 0.01), *norank_o__Clostridia_UCG-014* (R = 0.48, *P* = 0.01), and *Monoglobus* (R = 0.56, *P* < 0.01), while it was negatively correlated with *norank_f__Muribaculaceae* (R = −0.51, *P* < 0.01), *unclassified_f__Lachnospiraceae* (R = −0.48, *P* = 0.01), and *Rikenellaceae_RC9_gut_group* (R = −0.38, *P* = 0.04). Butanoic acid was positively correlated with *NK4A214_group* (R = 0.47, *P* = 0.01), *Monoglobus* (R = 0.38, *P* = 0.04), and *Christensenellaceae_R-7_group* (R = 0.45, *P* = 0.01). Similarly, isovaleric acid exhibited positive correlations with *NK4A214_group* (R = 0.58, *P* < 0.01), *norank_f__Ruminococcaceae* (R = 0.38, *P* = 0.04), *Akkermansia* (R = 0.40, *P* = 0.03), *norank_o__Clostridia_UCG-014* (R = 0.39, *P* = 0.03), and *Monoglobus* (R = 0.50, *P* = 0.01), but negative correlations with *norank_f__Muribaculaceae* (R = −0.49, *P* = 0.01), *Alistipes* (R = −0.39, *P* = 0.03), and *unclassified_f__Lachnospiraceae* (R = −0.38, *P* = 0.04). In addition, valeric acid was positively correlated with *NK4A214_group* (R = 0.52, *P* < 0.01), *Monoglobus* (R = 0.49, *P* = 0.01), and *norank_f__Eubacterium_coprostanoligenes_group* (R = 0.45, *P* = 0.01).

## DISCUSSION

This study provides a comprehensive multi-omics framework to elucidate how yaks orchestrate physiological, metabolic, and microbial adaptations to cope with the hypoxic and nutritional challenges of high-altitude environments. By integrating serum biochemistry, antioxidant and immune parameters, endocrine factors, plasma metabolomics, and gut microbiota profiles across the LA, MA, and HA groups, we demonstrate that altitude triggers a coordinated reprogramming of host metabolism and microbial ecology. The results support an adaptive model of energy reallocation under hypoxic stress, whereby yaks shift resources from growth and protein anabolism toward maintenance, antioxidation, and immune defense to sustain homeostasis in extreme environments.

Serum biochemical indicators provide a sensitive reflection of the metabolic and nutritional status of yaks living under environmental stress (12). In our study, yaks at HA exhibited significantly lower serum total protein, ALP, and BUN, consistent with attenuated anabolic metabolism and limited nutrient intake at high altitude (13, 14). The decline in BUN—a byproduct of amino acid catabolism—indicates a nitrogen-sparing strategy aimed at reducing protein degradation and conserving nitrogen under dietary restriction (15). Similarly, the decrease in ALT activity across MA and HA suggests impaired hepatic amino acid metabolism and reduced protein synthesis efficiency (16). Collectively, these alterations indicate that yaks at higher altitudes reduce anabolic metabolism to prioritize essential maintenance functions over growth. The endocrine data reinforce this interpretation. Levels of GH, IGF-1, and GHRH decreased progressively with altitude, while GHIH increased, reflecting suppression of the GH/IGF-1 axis under hypoxic conditions. This endocrine suppression is consistent with the energy reallocation hypothesis, in which animals redirect metabolic energy from growth to survival-related processes, such as oxygen transport and stress resistance (17). GH and IGF-1 are pivotal regulators of growth, nutrient metabolism, and oxidative balance (18, 19); their downregulation, coupled with enhanced inhibitory signaling, represents a strategic adaptation to limited energy availability in the HA environment. Similar patterns have been reported in high-altitude mammals, where hypoxia induces cortisol secretion, gluconeogenesis, and inhibition of growth-related pathways (17). Thus, the endocrine remodeling observed here exemplifies a trade-off between growth and homeostasis, a hallmark of chronic hypoxic adaptation.

High-altitude hypoxia increases mitochondrial ROS production during intermittent reoxygenation, imposing oxidative stress on tissues (20). Our data revealed altitude-dependent modulation of the antioxidant defense system. In the MA and HA groups, T-SOD and T-AOC were significantly elevated, whereas GSH-Px activity declined. This pattern suggests that oxidative stress intensifies with altitude, prompting upregulation of primary antioxidant defenses (SOD, T-AOC) while secondary defenses (GSH-Px) may be constrained by selenium deficiency or excessive utilization (21, 22). Enhanced T-SOD and T-AOC activity help neutralize superoxide radicals, maintaining cellular redox balance and protecting lipids and proteins from peroxidation damage (20). Such enzyme adjustments have been widely observed in plateau animals as adaptive strategies to mitigate hypoxia- and UV-induced oxidative injury (4). Increased antioxidant activity at high altitude indicates that yaks possess an intrinsic redox flexibility, allowing them to fine-tune enzyme systems to maintain redox homeostasis despite fluctuating oxygen availability. The decrease in GSH-Px may represent a compensatory redistribution of enzymatic roles, where SOD assumes greater importance in detoxifying superoxide radicals. Together, these findings suggest that antioxidant enhancement is an essential component of yak adaptation, enabling long-term survival under oxidative pressure in the Qinghai-Tibet Plateau.

The immune system is another key target of altitude adaptation, balancing pro-inflammatory activation for defense with anti-inflammatory regulation to prevent tissue damage. In the MA and HA groups, serum levels of immunoglobulins (IgA, IgM, IgG) and pro-inflammatory cytokines (IL-2, IL-6, TNF-α, IFN-γ) were significantly elevated, while anti-inflammatory cytokines (IL-4, IL-10) declined. Elevated immunoglobulins reflect enhanced humoral immune responses, possibly as protection against cold stress and hypoxia-related infections (4, 6). The simultaneous rise in IL-2, IL-6, TNF-α, and IFN-γ indicates activation of Th1-type immune pathways and macrophage function (23, 24), which strengthen host defense and tissue repair. Conversely, the suppression of IL-4 and IL-10 suggests reduced anti-inflammatory control. Under severe environmental stress, the body may transiently favor pro-inflammatory responses to eliminate pathogens and repair hypoxia-induced tissue injury (25, 26). IL-6, in particular, serves dual roles in inflammation and erythropoiesis by activating HIF-1/EPO signaling (27, 28), contributing to improved oxygen transport. Therefore, this cytokine profile represents a regulated pro-inflammatory state characteristic of highland adaptation. Although prolonged inflammation could be harmful, long-term high-altitude residents, such as yaks, likely achieve equilibrium between immune activation and tolerance. This immune rebalancing ensures effective immune surveillance without triggering chronic inflammation.

Untargeted plasma metabolomics revealed significant altitude-driven enrichment of multiple metabolic pathways. At MA, retinol metabolism was the most prominent pathway, suggesting its involvement in redox regulation and immune modulation under moderate hypoxia. Retinol and retinoic acid maintain epithelial integrity, regulate antioxidant gene expression, and modulate HIF signaling (29, 30). Enhanced retinol metabolism may therefore reinforce antioxidant defense and mucosal protection, helping yaks sustain tissue function in oxygen-poor environments (31). At HA, α-linolenic acid (ALA) metabolism and the TCA cycle were notably enriched. ALA, an essential omega-3 fatty acid, contributes to membrane fluidity, anti-inflammatory responses, and efficient energy production (9, 32). Its increased metabolism suggests adaptive lipid remodeling to sustain mitochondrial performance under hypoxia. Meanwhile, enrichment of the TCA cycle indicates intensified flux through core energy pathways, potentially reflecting accumulation of intermediates, such as succinate, which stabilizes HIF-1α and activates hypoxia-response genes (32, 33). Enhanced TCA and branched-chain amino acid (valine, leucine, isoleucine) metabolism may improve ATP generation efficiency and support nitrogen balance under oxygen limitation (9, 34). These findings demonstrate a metabolic flexibility that enables yaks to switch between lipid oxidation, amino acid turnover, and glycolysis to maintain energetic homeostasis across altitudes. Notably, alterations in glycosaminoglycan biosynthesis (heparan sulfate/heparin) were detected, indicating adjustments in cell signaling, tissue repair, and extracellular matrix remodeling (35, 36). These modifications may enhance vascular stability and protect against hypoxia-induced tissue injury, further supporting physiological adaptation. Overall, the metabolomic reprogramming of yaks reveals an integrated network linking antioxidant defense, energy metabolism, and oxygen signaling, enabling resilience to extremely high-altitude conditions.

The gut microbiota plays a crucial role in nutrient acquisition and immune regulation, and its composition was profoundly shaped by altitude. 16S rRNA analysis revealed that microbial α-diversity (*Chao1*, Shannon) and fermentative capacity (VFA concentrations) declined with altitude, indicating a less diverse and functionally constrained community. Such reductions reflect environmental filtering by hypoxia, cold, and limited forage diversity (9, 37). The MDI increased in the MA and HA groups, suggesting decreased microbial stability and a shift toward stress-tolerant taxa. Taxonomic analysis revealed altitude-dependent shifts in key microbial groups. *UCG-005* and *Rikenellaceae_RC9_gut_group*, belonging to Bacteroidetes, declined with altitude, potentially due to reduced availability of plant polysaccharides (37, 38). In contrast, *Christensenellaceae_R-7_group* and *Monoglobus* increased, indicating selective enrichment of taxa associated with host metabolic efficiency and fiber degradation (17, 39). *Christensenellaceae* has been linked to host leanness, metabolic health, and heritable microbiome traits, while *Monoglobus* specializes in pectin utilization, supporting carbohydrate fermentation (39). The rise of *norank_f__Ruminococcaceae* in HA yaks likely enhances VFA synthesis, compensating partially for reduced fermentative output. Consistent with these compositional changes, fecal VFAs (acetate, propionate, butyrate) declined significantly with altitude, indicating diminished fiber fermentation and energy harvest (40). VFAs are critical energy substrates for ruminants and also contribute to intestinal barrier function and immune regulation (41). The observed decline may thus contribute to lower nutrient absorption and altered immune homeostasis at high altitude. Moreover, BugBase functional prediction revealed a decrease in anaerobic taxa and an increase in mobile genetic elements, suggesting microbial genomic adaptation to environmental stress.

Spearman correlation analysis further revealed tight associations among plasma metabolites, fecal VFAs, and dominant microbial genera. In particular, acetic and propionic acids were positively correlated with *Christensenellaceae_R-7_group* and *Monoglobus*, whereas *Rikenellaceae_RC9_gut_group* showed a negative association. These relationships suggest that beneficial fermenters contribute to efficient fiber degradation and energy extraction, while altitude-enriched taxa may reflect metabolic stress or alternative fermentation routes. Similar metabolite–microbiota correlations have been reported in other highland ruminants, where specific taxa drive short-chain fatty acid production and energy yield under forage restriction (42–44). Such coordinated changes underscore a functional microbiome–metabolome interface that bridges host nutrient metabolism and microbial activity under hypoxia. Integrating these correlations with the observed serum biochemical and metabolomic profiles provides mechanistic support for the proposed serum–metabolome–microbiota axis in yak high-altitude adaptation.

Together, our findings delineate a tightly coupled host–microbiota system that enables yaks to maintain physiological equilibrium across altitudinal gradients. The reduction in protein metabolism, suppression of the GH/IGF-1 axis, enhancement of antioxidant defenses, and pro-inflammatory immune adjustment collectively form a host-level energy conservation strategy. Simultaneously, metabolic remodeling through retinol, ALA, and TCA pathways optimizes oxygen utilization and antioxidant capacity. The microbiota mirrors these host adaptations: declines in Bacteroidetes and enrichment of *Christensenellaceae* and *Monoglobus* reflect a community transition toward efficient fermentation under nutrient scarcity. This integrated framework extends previous observations that grazing and diet influence yak metabolic and microbial profiles by identifying altitude as an additional regulatory force shaping systemic adaptation (8).

The adaptive responses observed in yaks embody a finely tuned trade-off between survival and productivity under chronic hypoxia. Reduced anabolic metabolism and microbial fermentation efficiency conserve energy but limit growth potential at extreme altitudes. These findings highlight potential avenues for nutritional and microbial interventions. Supplementation with protein-rich and antioxidant feeds or probiotic strategies targeting *Christensenellaceae* and *Monoglobus* could enhance nutrient utilization and oxidative resilience in plateau herds (17, 39). Furthermore, selective breeding for individuals exhibiting superior antioxidant capacity, immune balance, and fermentation efficiency could promote herd health and sustainable adaptation. Future research should move beyond correlation toward causal validation of host–microbiota interactions under hypoxia. Controlled translocation or hypobaric simulation experiments, combined with host genomics and functional microbiome assays (e.g., bacterial isolation, metatranscriptomics, or fecal microbiota transplantation), could uncover molecular mechanisms and adaptive biomarkers (31, 40). Investigating how dietary modulation of retinol, ALA, and other key metabolites affects adaptation will provide practical guidance for precision feeding strategies. Collectively, these findings emphasize that yak high-altitude resilience arises from the synergistic regulation of host metabolism and gut microbiota, offering a scientific foundation for improved health management, selective breeding, and sustainable yak production on the Qinghai-Tibet Plateau.

### Conclusion

This study provides an integrated view of how yaks achieve physiological and metabolic adaptation to high-altitude environments. With increasing altitude, yaks exhibited reduced protein metabolism and suppressed growth-related endocrine activity, accompanied by enhanced antioxidant defenses and immune activation. Metabolomic analysis revealed distinct shifts in energy and lipid metabolism, particularly through retinol and α-linolenic acid pathways that support oxidative balance and cellular protection under hypoxia. The gut microbiota also underwent compositional and functional reorganization, favoring taxa associated with efficient fermentation and host metabolic stability. Together, these results indicate that yak adaptation to high altitude is driven by a coordinated host–microbiota response that reallocates energy from growth toward maintenance, antioxidation, and immune resilience. Understanding this serum–metabolome–microbiota network offers valuable insights for developing microbiome- and nutrition-based strategies to enhance yak health, performance, and sustainability on the Qinghai-Tibet Plateau.

## Supplementary Material

Reviewer comments

## Data Availability

The raw sequencing data are available in the NCBI SRA database under the BioProject accession number PRJNA1254471. For more details, please refer to the project page: ID 1254471—BioProject—NCBI.

## SUPPLEMENTAL MATERIAL

Fig. S1 spectrum.02549-25-s0001.tif Differential relative abundances of microbial taxa at the phylum and genus levels.

Supplemental material spectrum.02549-25-s0002.docx Supplemental figure legend.

OPEN PEER REVIEW reviewer-comments.pdf An accounting of the reviewer comments and feedback.

ASM does not own the copyrights to Supplemental Material that may be linked to, or accessed through, an article. The authors have granted ASM a non-exclusive, world-wide license to publish the Supplemental Material files. Please contact the corresponding author directly for reuse.

## References

[1] Ma J, Zhu Y, Wang Z, Yu X, Hu R, Wang X, Cao G, Zou H, Shah AM, Peng Q, Xue B, Wang L, Zhao S, Kong X. 2020. Comparing the bacterial community in the gastrointestinal tracts between growth-retarded and normal yaks on the qinghai-tibetan plateau. Front Microbiol 11:600516. doi:10.3389/fmicb.2020.600516 33391217 PMC7775487

[2] Ji Q-M, Xin J-W, Chai Z-X, Zhang C-F, Dawa Y, Luo S, Zhang Q, Pingcuo Z, Peng M-S, Zhu Y, Cao H-W, Wang H, Han J-L, Zhong J-C. 2021. A chromosome-scale reference genome and genome-wide genetic variations elucidate adaptation in yak. Mol Ecol Resour 21:201–211. doi:10.1111/1755-0998.13236 32745324 PMC7754329

[3] Suo H, Zhao X, Qian Y, Sun P, Zhu K, Li J, Sun B. 2016. Lactobacillus fermentum Suo attenuates HCl/Ethanol Induced Gastric Injury in Mice through its antioxidant effects. Nutrients 8:155. doi:10.3390/nu8030155 26978395 PMC4808883

[4] Bai J, Li L, Li Y, Zhang L. 2022. Genetic and immune changes in Tibetan high-altitude populations contribute to biological adaptation to hypoxia. Environ Health Prev Med. doi:10.1265/ehpm.2200040 PMC9640738 36244759

[5] Vargas A, Mkrtchyan K, Penuelas V, Pham K, Frost S, Moya EA, Yu JJ. 2024. Acute exposure to high-altitude results in changes to immune cell populations. Physiology (Bethesda) 39. doi:10.1152/physiol.2024.39.S1.2515

[6] Pham K, Vargas A, Frost S, Shah S, Heinrich EC. 2024. Changes in immune cell populations during acclimatization to high altitude. Physiol Rep 12:e70024. doi:10.14814/phy2.70024 39551933 PMC11570420

[7] Mishra KP, Ganju L. 2010. Influence of high altitude exposure on the immune system: a review. Immunol Invest 39:219–234. doi:10.3109/08820131003681144 20380520

[8] Xie Y, Cidan Y, Cisang Z, Gusang D, Danzeng Q, Basang W, Zhu Y. 2025. Effects of warm-season feeding on yak growth, antioxidant capacity, immune function, and fecal microbiota. Microbiol Spectr 13:e01001–01025. doi:10.1128/spectrum.01001-25 PMC12323587 40548706

[9] Liu D, Chen D, Xiao J, Wang W, Zhang L-J, Peng H, Han C, Yao H. 2024. High-altitude-induced alterations in intestinal microbiota. Front Microbiol 15:1369627. doi:10.3389/fmicb.2024.1369627 38784803 PMC11111974

[10] Yu W, Yang J, Teng L-W, Zhao X-L, Zhu Z-Y, Cui S, Du W-G, Liu Z-S, Zeng Z-G. 2025. Reciprocal translocation experiments reveal gut microbiome plasticity and host specificity in a Qinghai-Xizang Plateau lizard. Zool Res 46:139–151. doi:10.24272/j.issn.2095-8137.2024.284 39846192 PMC11891006

[11] Kanehisa M, Furumichi M, Tanabe M, Sato Y, Morishima K. 2017. KEGG: new perspectives on genomes, pathways, diseases and drugs. Nucleic Acids Res 45:D353–D361. doi:10.1093/nar/gkw1092 27899662 PMC5210567

[12] Ahmat M, Cheng J, Abbas Z, Cheng Q, Fan Z, Ahmad B, Hou M, Osman G, Guo H, Wang J, Zhang R. 2021 Effects of Bacillus amyloliquefaciens LFB112 on growth performance, carcass traits, immune, and serum biochemical response in broiler chickens. Antibiotics (Basel) 10:1427. doi:10.3390/antibiotics10111427 34827365 PMC8614806

[13] Onyeneke E, Njoya H, Ebot M, Uchungo G, Eriyamremu G, Alumanah E. 2004. Liver function status in some nigerian children with protein energy malnutrition. Orient J Med 15. doi:10.4314/ojm.v15i1.29048

[14] Jain A, Jadhav AA, Varma M. 2013. Relation of oxidative stress, zinc and alkaline phosphatase in protein energy malnutrition. Arch Physiol Biochem 119:15–21. doi:10.3109/13813455.2012.737809 23373727

[15] Zhang X, Cao Z, Yang HC, Wang Y, Wang W, Li S. 2024. Effect of high altitude on serum biochemical parameters, immunoglobulins, and rumen metabolism of sanhe heifers. Fermentation 10:170. doi:10.3390/fermentation10030170

[16] Gokulakrishnan M, Kumar R, Pillai BR, Nanda S, Bhuyan SK, Kumari R, Debbarma J, Ferosekhan S, Siddaiah GM, Sundaray JK. 2022. Dietary brewer’s spent yeast enhances growth, hematological parameters, and innate immune responses at reducing fishmeal concentration in the diet of climbing perch, Anabas testudineus fingerlings. Front Nutr 9:982572. doi:10.3389/fnut.2022.982572 36159475 PMC9490235

[17] Storz JF. 2007. Hemoglobin function and physiological adaptation to hypoxia in high-altitude mammals. J Mammal. doi:10.1644/06mamms199r1.1

[18] Hidalgo AJM, Rio-Moreno MD, Pérez-Gómez JM, Luque RM, Kineman RD. 2025. Update on regulation of GHRH and its actions on GH secretion in health and disease. Rev Endocr Metab Disord. doi:10.1007/s1115402509943y 39838154

[19] Bioletto F, Varaldo E, Gasco V, Maccario M, Arvat E, Ghigo E, Grottoli S. 2024. Central and peripheral regulation of the GH/IGF-1 axis: GHRH and beyond. Rev Endocr Metab Disord. doi:10.1007/s11154024099336 39579280

[20] Huang M, Zhang X, Yan W, Liu J, Wang H. 2022. Metabolomics reveals potential plateau adaptability by regulating inflammatory response and oxidative stress-related metabolism and energy metabolism pathways in yak. J Anim Sci Technol 64:97–109. doi:10.5187/jast.2021.e129 35174345 PMC8819316

[21] Zhang X, Huang S, Li S, Wang W. 2022. Effects of altitude on the digestion performance, serum antioxidative characteristics, rumen fermentation parameters, and rumen bacteria of sanhe heifers. Front Microbiol 13:875323. doi:10.3389/fmicb.2022.875323 35572662 PMC9097872

[22] Rytz CL, Pun M, Mawhinney JA, Mounsey CA, Mura M, Martin A, Pialoux V, Hartmann SE, Furian M, Rawling JM, Lopez I, Soza D, Moraga FA, Lichtblau M, Bader PR, Ulrich S, Bloch KE, Frise MC, Poulin MJ. 2022. Differential effects of high-altitude exposure on markers of oxidative stress, antioxidant capacity, and iron profiles. Am J Physiol Regul Integr Comp Physiol 323:R445–R456. doi:10.1152/ajpregu.00321.2021 35938686

[23] Hasnat MA, Cheang I, Dankers W, Lee JP, Truong LM, Pervin M, Jones SA, Morand EF, Ooi JD, Harris J. 2022. Investigating immunoregulatory effects of myeloid cell autophagy in acute and chronic inflammation. Immunol Cell Biol 100:605–623. doi:10.1111/imcb.12562 35652357 PMC9542007

[24] Yin J, Lv J, Yang S, Wang Y, Huang Z, Wang X, Hou G, Zhou W, Liu Y, Wang W, et al.. 2025. Multi-omics reveals immune response and metabolic profiles during high-altitude mountaineering. Edited by J Yin, J. Lv, S. Yang, Y. Wang, Z. Huang, X. Wang, G. Hou, W. Zhou, Y. Liu, W. Wang, X. Lin, Y. Huang, Y. Zheng, C. Wei, Y. Yuan, Y. Huang, C. Liu, H. Tao, H. Liu, R. Liu, and J. Wang. Cell Rep 44:115134. doi:10.1016/j.celrep.2024.115134 39932189

[25] Aramwit P, Luplertlop N, Kanjanapruthipong T, Ampawong S. 2018. Effect of urea-extracted sericin on melanogenesis: potential applications in post-inflammatory hyperpigmentation. Biol Res 51:54. doi:10.1186/s4065901802045 30497518 PMC6262948

[26] Wang H, Li R, Rao Y, Liu S, Hu C, Zhang Y, Meng L, Wu Q, Ouyang Q, Liang H, Qin M. 2022. Enhancement of the bioavailability and anti-inflammatory activity of glycyrrhetinic acid via novel soluplus - a glycyrrhetinic acid solid dispersion. Pharmaceutics 14:1797. doi:10.3390/pharmaceutics14091797 36145545 PMC9504515

[27] Hartmann G, Tschöp M, Fischer R, Bidlingmaier C, Riepl R, Tschöp K, Hautmann H, Endres S, Toepfer M. 2000. High altitude increases circulating interleukin-6, interleukin-1 receptor antagonist and C-reactive protein. Cytokine 12:246–252. doi:10.1006/cyto.1999.0533 10704252

[28] Pham K, Parikh K, Heinrich EC. 2021. Hypoxia and inflammation: insights from high-altitude physiology. Front Physiol 12:676782. doi:10.3389/fphys.2021.676782 34122145 PMC8188852

[29] Cyr A, Kohut L, Chambers L, Stratimirovic S, Zuckerbraun B. 2021. Circulating metabolomic analysis following cecal ligation and puncture in young and aged mice reveals age-associated temporal shifts in nicotinamide and histidine/histamine metabolic pathways. Oxid Med Cell Longev 2021:5534241. doi:10.1155/2021/5534241 34512866 PMC8433009

[30] Wang Hai-meng, Wu C, Jiang Y, Wang W, Jin H. 2020. Retinol and vitamin A metabolites accumulate through RBP4 and STRA6 changes in a psoriasis murine model. Nutr Metab (Lond) 17:5. doi:10.1186/s12986-019-0423-y 31956331 PMC6958599

[31] Xu H, Ma Z, Lu J, Li Y, Li Q, Yin S, He H, Xiong Y, Xiong X, Li J, Lan D, Fu W. 2024. Integrative analysis of transcriptome and metabolome reveals the heterogeneity of ovarian follicles between high-altitude Yak (Bos grunniens) and Low-altitude cattle (Bos taurus). Livest Sci 289:105557. doi:10.1016/j.livsci.2024.105557

[32] Guo T, Wang X, Zhang Q, Jia Y, Liu H, Hu L, Zhao N, Xu S, Duan Y, Jia K. 2024. Transcriptomics and metabolomics insights into the seasonal dynamics of meat quality in yak on the Qinghai-Tibetan Plateau. BMC Genomics 25:1194. doi:10.1186/s12864024110935 39695977 PMC11654168

[33] Atallah R, Olschewski A, Heinemann A. 2022. Succinate at the crossroad of metabolism and angiogenesis: roles of SDH, HIF1α and SUCNR1. Biomedicines 10:3089. doi:10.3390/biomedicines10123089 36551845 PMC9775124

[34] Ma X, Duan C, Wang X, Tao Y, Yang L, Teng Y, Pan Y, Zhang M, Xu J, Sheng J, Wang X, Jin P. 2025. Human gut microbiota adaptation to high-altitude exposure: longitudinal analysis over acute and prolonged periods. Microbiol Spectr 13:e0291624. doi:10.1128/spectrum.02916-24 40257273 PMC12131729

[35] Sieme D, Griesinger C, Rezaei-Ghaleh N. 2022. Metal binding to sodium heparin monitored by quadrupolar NMR. Int J Mol Sci 23:13185. doi:10.3390/ijms232113185 36361973 PMC9655979

[36] Tanino Y, Wang X, Nikaido T, Misa K, Sato Y, Togawa R, Kawamata T, Kikuchi M, Frevert CW, Tanino M, Kojima T, Shibata Y. 2019. Syndecan-4 inhibits the development of pulmonary fibrosis by attenuating TGF-β signaling. Int J Mol Sci 20:4989. doi:10.3390/ijms20204989 31600983 PMC6834137

[37] Wang X, Zhang Z, Li B, Hao W, Yin W, Ai S, Han J, Wang R, Duan Z. 2022. Depicting fecal microbiota characteristic in yak, cattle, yak-cattle hybrid and tibetan sheep in different eco-regions of qinghai-tibetan plateau. Microbiol Spectr 10:e0002122. doi:10.1128/spectrum.00021-22 35863031 PMC9430443

[38] Li L, Li K, Bian Z, Chen Z, Li B, Cui K, Wang F. 2022. Association between body weight and distal gut microbes in Hainan black goats at weaning age. Front Microbiol 13:951473. doi:10.3389/fmicb.2022.951473 36187995 PMC9523243

[39] Waters JL, Ley RE. 2019. The human gut bacteria Christensenellaceae are widespread, heritable, and associated with health. BMC Biol 17:83. doi:10.1186/s12915-019-0699-4 31660948 PMC6819567

[40] He S, Zhao S, Wang Z, Dai S, Mao H, Wu D. 2024. Impact of seasonal variation in pasture on rumen microbial community and volatile fatty acids in grazing yaks: insights from high-altitude environments. Microorganisms 12:1701. doi:10.3390/microorganisms12081701 39203543 PMC11357337

[41] Shaopeng C, Changze C, Youpeng Q, Baohong M, Meixian Z, Chenyue J, Chune Z, Xiangyan W, Jiang H, Bingang S, Xueming M, Zhidong Z, Xiaolan Z. 2024. Studies on fatty acids and microbiota characterization of the gastrointestinal tract of Tianzhu white yaks. Front Microbiol 15:1508468. doi:10.3389/fmicb.2024.1508468 39895933 PMC11784337

[42] Wang X, Zhang Q, Guo T, Li S, Jia Y, Xu S. 2025. Multi-omics analysis reveals host-microbe interactions driving divergent energy allocation strategies in Tibetan sheep under cold-season feeding regimes. J Anim Sci Biotechnol 16:122. doi:10.1186/s40104-025-01259-w 40890867 PMC12403966

[43] Yang X, Fan X, Jiang H, Zhang Q, Zhang Q, Dang S, Long R, Huang X. 2022. Simulated seasonal diets alter yak rumen microbiota structure and metabolic function. Front Microbiol 13:1006285. doi:10.3389/fmicb.2022.1006285 36212853 PMC9538157

[44] Cui X, Wang Z, Guo P, Li F, Chang S, Yan T, Zheng H, Hou F. 2023. Shift of feeding strategies from grazing to different forage feeds reshapes the rumen microbiota to improve the ability of tibetan sheep (Ovis aries) to adapt to the cold season. Microbiol Spectr 11:e0281622. doi:10.1128/spectrum.02816-22 36809032 PMC10100778

