# Supplementary material for: Effect of altitudes on serum parameters, metabolome, and gut microbiota in yaks on the Qinghai-Tibet Plateau
Source: Microbiol Spectr. 2025 Dec 18;14(2):e02549-25. doi: 10.1128/spectrum.02549-25 (PMC12889084; doi:10.1128/spectrum.02549-25)
Supplement: Supplemental material — Supplemental figure legend. [file spectrum.02549-25-s0002.docx]

**Figure S1** Differential relative abundances of microbial taxa at the phylum and genus levels: (A) *Verrucomicrobiota*; (B) *Actinobacteriota*; (C) *UCG-005*; (D) *Rikenellaceae_RC9_gut_group*; (E) *norank_f__Eubacterium_coprostanoligenes_group*; (F) *Christensenellaceae_R-7_group*; (G) *Monoglobus*; (H) *norank_f__UCG-010*; (I) *Akkermansia*; (J) *Alistipes*; (K) *Prevotellaceae_UCG-004*; (L) *norank_f__Ruminococcaceae*; (M) *norank_f__Muribaculaceae*; (N) *Romboutsia*; (O) *NK4A214_group*.

^a – c^ Values within a row with no common superscripts differ significantly (*P* < 0.05).
